# Study protocol: a randomized control trial of African American families fighting parental cancer together

**DOI:** 10.1186/s12885-018-5052-8

**Published:** 2018-11-20

**Authors:** Nicole S. McKinney, Shannon Virtue, Frances Marcus Lewis, Alliric I. Willis, Tanisha Pettyjohn, La-Rhonda Harmon, Adam Davey

**Affiliations:** 1 School of Nursing, University of Delaware, 381 McDowell Hall, 25 N. College Ave, Newark, DE 19716 USA; 2 Helen F. Graham Cancer Center & Research Institute, Christiana Care Health System, 4701 Ogletown-Stanton Road, Suite 2200, Newark, DE 19713 USA; 3 University of Washington School of Nursing, Box 357262, 1959 NE Pacific St, Seattle, WA 98195 USA; 4 Jefferson University, 1100 Walnut Street, 5th Floor, Philadelphia, PA 19107 USA; 5 University of Delaware, 381 McDowell Hall, 25 N. College Ave, Newark, DE 19716 USA; 6 The Ladino Group, 3900 Ford Road, Philadelphia, PA 19131 USA; 7 Department of Behavioral Health and Nutrition, University of Delaware, 016 Carpenter Building, 26 N. College Ave, Newark, DE 19716 USA

**Keywords:** African Americans, cancer., adolescent., solid tumor., intervention groups., randomized control trial.

## Abstract

**Background:**

African American adults experience a disproportionate burden and increased mortality for most solid tumor cancers and their adolescent children are negatively impacted by the illness experience. The purpose of this randomized clinical trial is to evaluate the efficacy of a culturally sensitive family-based intervention program developed for African American families coping with solid tumor parental cancer using an intention-to-treat approach. Primary outcome is adolescent depressive symptoms at end of treatment.

**Methods:**

A sample of 172 African American families will be enrolled from two diverse oncology centers (Helen Graham Cancer Center in Newark, DE, and Kimmel Cancer Center in Philadelphia, PA). Eligible families will be randomized either to a 5-session intervention Families Fighting Cancer Together (FFCT) or a 5-session parent-only psycho-educational (PED) program. Assessments will occur at weeks 0 (baseline), 8 (end-of-treatment), 24, and 52.

**Discussion:**

Treatments to help African American adolescents cope with the impact of parental cancer are scarce and urgently needed. If successful, this proposed research will change the nature of intervention support options available to African Americans, who are overrepresented and underserved by existing services or programs.

**Trial registration:**

This project is registered with ClinicalTrials.gov (Protocol #: NCT03567330).

## Background

In 2018, an estimated 1.7 million newly adult cancer cases will be diagnosed in the United States [1]. Breast cancer, lung, prostate and colorectal cancer are among some of the most common cancer cases projected in 2018 [2]. Of these 1.7 million adults new diagnosed with cancer in 2018, nearly 800,000 children 18 years old or younger will be affected by a parent’s cancer diagnosis [2]. Moreover, three million children under the age of 18 face the devastating challenge of coping with a parent who is living with cancer [2]. Parental cancer is associated with significant emotional distress in both diagnosed parents and their children [3–5]. In addition, many parents diagnosed with cancer experience high rates of depressed mood for two or more years post their initial diagnosis [6, 7]. In some cases, parents with cancer have an absence of depressed mood, although in most incidences parents diagnosed with parental cancer face treatment demands and preoccupation with cancer. This can lead to these parents with cancer more likely to be physically and emotionally unavailable to their children in the home [5, 8–10]. Consequently, these children whose parents have cancer, an estimated 22–33% of these children are more likely to display clinical levels of emotional distress, particularly adolescents (ages 12–18) [11, 12].

African American adults experience a disproportionate burden for most solid tumor cancers (e.g., breast, prostate, colorectal, lung) and have higher cancer mortality rates than Whites [13]. African American women have a higher incidence of breast cancer and higher rates of mortality under the age of 50, when these women are more likely to be raising children [14]. Collectively, African American men and women have a higher incidence of colon cancer, while African American men have a higher incidence of prostate cancer at all ages [13, 15]. These facts raise concern for the African American children of these diagnosed parents with cancer being at a high risk for parental cancer-related distress. African Americans have not been well represented in intervention studies compared with White middle-class samples [16]. Despite the known burden of parental cancer on adolescents, the substantial under-representation of African Americans in the clinical trial literature, and the dominance of studies with primarily White parents and children, there have been no randomized clinical trial intervention studies to assist African American parents and their adolescents navigate the illness burden of the parent’s cancer. Prior research suggests African American patients tend not to volunteer for clinical trials because of understandable mistrust of providers and a perceived lack of cultural sensitivity and institutional as well as structural barriers to recruitment and participation [15–18]. Adaptations tailored to a specific ethnic/racial group are more effective than those adapted for a variety of minority groups [19]. Additionally, differences in attitudes, daily functioning, and levels of distress among different ethnic and racial groups are well-documented [18–22]. Many African Americans face stressors which can influence and exacerbate difficulty in coping with cancer, such as but not limited to: financial challenges, community violence, substandard educational system, single-parent households, racial discrimination, heath treatment disparities, urban blight, etc. [18–22] Thus, taking these barriers into consideration, this 5-session 8-week culturally sensitive intervention, African American Families Fight Cancer Together (FFCT) will be compared with Psychoeducation (PED).

Two theoretical frameworks were selected to support the direction of this research trial. Each attachment theory and social learning theory provide the theoretical foundation of the Families Fighting Cancer Together (FFCT) intervention model [23, 24]. Investigators in this research also borrowed perspectives regarding the treatment structure and content from Beardslee’s work with families coping with parental depression and Clarke’s work preventing depression and anxiety in adolescents [25, 26].

## Aims

### Primary aim

Aim 1. Compare the efficacy of FFCT to PED in reducing depressive symptoms among African American (AA) adolescents at post-treatment using an intention-to-treat (ITT) analysis.

### Secondary aim

Aim 2. Compare the efficacy of FFCT to PED in reducing parental stress among AA parents at post-treatment using ITT analysis.

### Exploratory aims

Aim 3a. Determine treatment differences in trajectories of adolescent depressive symptoms, adolescent anxiety, and parental stress from baseline to 12-month follow-up.

Aim 3b. Determine whether perceived levels of group support, adolescent gender and age, parent’s marital and socioeconomic status, and parent’s cancer staging modify the effects of treatment on adolescent depression and anxiety.

Aim 3c. Determine whether pre-post changes in parent-adolescent attachment and communication mediate the association between treatment and adolescent depressive symptoms and anxiety at 6- and 12-month follow-ups.

## Methods/design

### Study design

This randomized control trial (RCT), will be conducted to address the specific aims of this research. African American parents (i.e., caregivers) newly diagnosed in the past 12 months of stages I, II, or III solid tumor cancer (e.g.., breast, lung, prostate, colon) and who have at a least one of their adolescent children (ranging in age 12–18 years old) living at home will be randomized to one of two five-session treatments. Families Fighting Cancer Together (FFCT) will include parent and adolescent child(ren) participation and Psychoeducation (PED) consists on psycho-education for the parent only. The University of Delaware Institutional Review Board and participating recruitment sites have approved all study protocols. A list of study sites can be obtained from clinicaltrials.gov. Participating parents in the study will provide informed consent and their adolescent child(ren) will offer written assent. A target sample of *N =* 172 families will be recruited over the 5-year study.

### Statistical power

Sample size was determined from pilot study data for the primary outcome (child depressive symptoms on the CDI) and endpoint (post-treatment) using power = 8 and α = .05 [27]. For all other analyses, minimum detectable effects are presented for fixed sample size. Pilot data indicate a greater decrease in depressive symptoms from pre-treatment to post-treatment among children in the FFCT intervention compared with children receiving PED equivalent to an effect size of Cohen’s d = 0.38, and pre-post correlations of 0.68. This suggests that a sample size of 60 families per study arm will yield power ≥ .8 with α = .05. Conservatively assuming retention of 70% (retention in the single site pilot was 85%), this indicates that recruitment of 172 study participants will ensure adequate statistical power for testing the primary study aim.

For tests of differences at post-treatment, a total sample size of 120 is used. Minimum detectable effects (in SD units) vary as a function of the correlation between pre-treatment and post-treatment measures and range between 0.31 for a correlation of 0.8 and 0.51 for a correlation of 0.2. Test of differences at 12-month follow-up assume that 80% of participants at post-treatment are retained to follow-up but benefit statistically from two additional occasions of measurement. Minimum detectable effects (in SD units) vary as a function of the correlation between pre-treatment and follow-up measures and range between 0.28 for a correlation of 0.8 and 0.38 for a correlation of 0.2.

### Study setting

This study takes place in the Northeast region of the United States, in Newark, DE, and Philadelphia, PA.

### Study population

Participant eligibility for this study includes: (a) parents must identify as non-Hispanic African American cancer patients diagnosed for the first time with stage I, II, or III solid tumor cancer within the past 12 months and report being a parent or primary caregiver for at least one adolescent aged 12–18 years living at home who has been informed of the diagnosis; (b) the primary parent or caregiver is an adult living with and having responsibility for the adolescent child(ren); and (c) the diagnosed parent is defined as an adult who is a biological parent, stepparent, grandparent, or fictive kin.

Parent exclusion criteria include: (a) if the parent has a serious mental health illness (e.g., clinical depression as evidenced by the Center for Epidemiological Studies Depression Scale > 27); (b) has psychotic features or severe cognitive impairment; and (c) is not fluent in spoken English. Adolescent exclusion criteria include: (a) the adolescent is severely depressed (CDI > 25; recommended clinical cutoff) or anxious (RCMAS> 42; recommended clinical cutoff) at baseline; (b) evidence of psychotic features; (c) presence of cognitive impairment (e.g., mental retardation, severe developmental disorders) as evidenced by educational records, parental report and/or clinical impression; and (d) currently in active outpatient mental health treatment.

### Recruitment of participants

The target sample will be primarily recruited from the Helen Graham Cancer Center and community recruitment sites, with Kimmel Cancer Center of Jefferson University serving as a secondary recruitment site. Eligible AA patients will first be sent a letter, and then called up to twice to see if they are interested in the prevention program. If a patient expresses an interest in the study, he or she will first sign a release of information, so the recruiter can contact him or her. The recruiter will then mail out a recruitment letter and brochure and will then contact the patient by telephone to explain the study. If the patient continues to meet study inclusion criteria and his/her adolescent child wants to participate, the assessment team will schedule a face-to-face evaluation with parents and adolescents either at the oncology clinic, the patient’s home, or at the University of Delaware, depending on what the patient/family prefers.

Following informed consent during the intake meeting, the patient and adolescent will be fully screened for study inclusion criteria (e.g., screened for parental and adolescent depression/anxiety). If patients, family members, and adolescents are appropriate for the study, the recruiter will fully explain the study and participants will sign consent/assent forms. Families that are deemed ineligible during screening will be provided with an appropriate referral for additional services (at cost to them when applicable). Parents and adolescents will then separately fill out baseline assessments using a HIPAA-secured tablet. This process should take no longer than 60 min to complete.

### Randomization

Randomized permuted blocks will be used to allocate groups of parent-adolescent dyads to each study arm (FFCT or PED). The data manager will prepare complete sets of allocations which will be placed in sequentially numbered sealed opaque envelopes containing treatment assignments for each group of 5 to 8 families. Once 5 families are recruited, group allocation will be made after a week has elapsed without any additional family participants recruited or once 8 families have been recruited, whichever occurs first.

The assessment team will be blind to the allocation condition and know only the serial numbers. At randomization, following baseline assessments, only the program manager opens the envelope for the group’s allocation. This approach provides both dynamic balancing and prospective partial blinding of the project manager to treatment assignment. At the end of the study, unused sealed envelopes will be retained along with the log to assure that randomization has been carried out as assigned.

### Attrition

We will implement a variety of procedures to reduce attrition. At the baseline assessment, all families are informed of and agree to the follow-up assessments before they are randomized. As part of the intake assessments, all patients complete a form listing their home address, phone numbers, and at least 3 contact persons who will always know the family’s location. The locator information is updated at each data collection point and is an important tool for finding patients who move during the follow-up phase.

Families are scheduled for the next follow-up at the previous appointment and are reminded of their appointments by a series of phone calls and mailings. A letter thanking families for participating in the study is sent within 1 week of randomization, and reminder letters are sent 1 week before the assessment is scheduled. Confirmation telephone calls are made 24 h prior to a scheduled follow-up appointment. When necessary, staff will travel to families’ homes, Helen F. Graham Cancer Center, or other locations to complete assessments. We will also mail cards to each caregiver and adolescent on their birthdays and all major holidays. These cards will be initiated as soon as the family agrees to participate. The cards remind participants of their involvement in the project and enable the investigators to determine if a family has moved (e.g., card is returned). These retention procedures are standard practice and usually produce research retention rates of 85% or better. In order to help verify external validity of the final sample, we will track the number of families who make it to intake, randomization, and treatment. Refusers and dropouts will be documented, and we will call these families to gather information about barriers and facilitators to treatment.

To increase retention and attendance, we will provide money for public transportation, food, drinks, and an onsite babysitter to care for any younger children when families participate in the 5-session prevention program. Removing barriers for completing assessments are important when working with a lower income urban minority population. To increase the likelihood of completion of the 4 assessments (baseline, post-treatment, 6 and 12-month follow up), we will compensate adolescents and one parent each: $30 for 1 parent and $30 for adolescents at the baseline assessment; $35 for 1 parent and $35 for adolescents at the post-treatment assessment (week 8), $40 for 1 parent and $40 for adolescents at the 6-month follow-up, $40 for 1 parent and $40 for adolescents at the 12-month follow-up assessment.

### Therapist selection, training, supervising, and monitoring

Prior research suggests it is important to have 2 clinicians (at least master’s level educated) running the intervention together as co-facilitators, matching race of the participants to race of the group facilitators. There will be two different teams of therapists for each condition; the principal investigator (PI) will hire 1 licensed clinical supervisor and at least 3 master’s level educated AA therapists who will be trained to provide either FFCT or PED. The clinical supervisor will have at least a doctoral degree and 5 years of experience doing family-based therapy. The therapists will have at least 2 years of family therapy training or experience and at least a master clinical degree. The PI and project manager will train the clinical supervisor and therapists. Training will begin with reading the treatment manual and attending a training workshop. The workshop will focus on program content, including sensitivity to cultural and socioeconomic status differences. The PI will certify the therapists using the adherence measures; they must receive an average rating of 5 or higher (7-point scale). Once the trial begins, the clinical supervisor will meet with the therapists weekly to review their video recorded sessions and complete the fidelity checklist for FFCT and PED sessions. In years 4 and 5 of the study, objective raters will be trained to rate adherence. Twenty percent of the video-recordings consisting of early middle and late sessions in the treatment will be rated.

### Blind assessors

Several steps will be taken to ensure the assessment team and adherence raters are blind to treatment assignment, in accordance with Consort 2010 guidelines including: (1) The assessment team will not work with clinical staff and will not attend meetings in which participants’ treatment assignment or progress is discussed. (2) Interviewers will be trained to not discuss participants’ involvement in the project with the subject. (3) At the start of each interview, participants will be given a card asking them not to mention which treatment they received. These procedures should limit staff’s influence on participant report; any breaking of “blindness” will be documented and reported according to Consort guidelines.

### The treatment models: (FFCT) and (PED)

Families Fighting Cancer Together (FFCT) is a culturally sensitive manualized, empirically supported, brief (5 sessions; 1 session every other week over 8 weeks) family support group program designed to help African American (AA) families cope with the stress of solid tumor parental cancer. FFCT derives from an integration of the research team’s previous studies with racially diverse samples of parents coping with cancer and their adolescents [28, 8, 9, 22, 27].

FFCT is characterized by four key features that address the particular needs of low-to-middle income AA families: (1) Therapists focus heavily on engagement and reduction of logistical (e.g., financial and transportation barriers) as well as psychological barriers (e.g., stigma, treatment readiness, negative experience with the mental health system). (2) Caregiver is defined broadly to reflect the wide range of family structures common in AA families. (3) Intervention targets Afro-centric coping skills: (a) faith; (b) encouraging fictive kin instrumental and emotional support; (c) highlighting positives; and (d) encouraging flexibility in family roles. (4) The support group structure and emphasis on brief treatment with a specific problem focus is culturally congruent with AAs who tend not to prefer individual therapy because of the stigma associated with mental health treatment and who prefer an action-oriented therapeutic approach with short-term goals [18, 21, 29].

FFCT includes the following 5 sessions: Sessions 1 and 2 are 90-min adolescent support group sessions (up to 8 AA youth). Session 1 focuses on improving awareness and understanding about how their parents’ cancer is affecting them. The group facilitators focus on the promotion of empathy and understanding among group members, reducing tension, increasing fun activities, and maintaining normal routines. In session 2, the facilitators help adolescents develop adaptive coping skills and prepare youth to more openly communicate their thoughts, fears, and feelings to their parents about the cancer experience during sessions 4 and 5. Session 3 is a 2-h, parent-only support group session to help parents get ready to talk more openly to their adolescents during sessions 4 and 5. This session focuses on: (1) therapist alliance and trust building with parent(s); (2) improving parents’ self-care while navigating cancer; (3) teaching parents to listen and to validate their adolescent’s experiences so youth can more openly communicate fears and worries; and 4) promoting more open communication between parents and children using Afro-centric resilience. Sessions 4 and 5 includes two (2 h) interactive multiple family group sessions (up to 8 families). In session 4, group facilitators focus discussions on parental cancer and on the stress it places on families, in particular helping adolescents openly share their thoughts, feelings, and fears with parents and helping their parents to listen and validate them. Session 5 focuses on facilitating secure parent-child attachment, so adolescents can talk directly to their parents and parents can respond to their children’s worries/fears in a responsive and empathic way. Facilitators focus discussions during sessions 1 to 5 on faith, spirituality, resilience, and utilizing fictive kin and the African American community (e.g., the church) for practical, emotional, and spiritual support [30].

Psychoeducation (PED) consists of only parents receiving a 5-session (1 session every 2 weeks over 8 weeks) psycho-education group, which was adapted from an American Cancer Society (ACS) free online educational program [31]. This program, (previously known as *I Can Cope*) was developed for cancer patients, families, and friends. This standardized online program included self-paced lectures about cancer: managing treatments and side effects; eating healthily during and after treatment; and finding resources. Explicit permission was attained from ACS to use this program. The purpose for choosing this program as the PED comparison group was it closely resembled what oncology clinics offer to adult cancer patients [32]. The ACS’s structured PowerPoint lectures and videos from *I Can Cope* have recently been updated and organized differently. Group facilitators will cover the following in 5 (1-h) psycho-education sessions with the most current ACS informational materials: (1) managing effects of illness/treatment; (2) nutrition during treatment; (3) mobilizing resources/support; (4) addressing self-esteem/intimacy; and (5) integrating mind, body, and spirit. This comparison group controls for contact time with group facilitators, filling out baseline information, posttest, 6- and 12-month follow-up assessments; it also restricts the focus on parent-child communication and attachment in the experimental group (FFCT).

#### Treatment Fidelity

Standardized fidelity treatment checklists were developed for FFCT and PED to monitor treatment integrity and dosage. These treatment checklists were tested in our previous pilot studies [22, 27]. Monitoring involves both dosage and purity; dosage of the intervention is measured by the completion of the number of sessions. Purity involves monitoring the extent to which the program was delivered as designed. All sessions will be video-recorded. Once the trial begins, the clinical supervisor will meet separately with the two teams of therapists weekly to review their video-recorded sessions, complete the fidelity checklist for both FFCT and PED sessions, and re-train as necessary to monitor treatment fidelity. Second, a random sample of 25% of all group sessions will later be rated by 2 coders blind to treatment assignment on the same checklist in years 4 and 5. With a total of 172 families (86 families/arm) who attend 5 (90-min to 2-h) support group sessions, it is estimated there will be 14 FFCT groups with 5 to 8 families/group and 14 PED groups with 5 to 8 families/group run during the course of this study. All intervention sessions will be video recorded using a HIPAA-compliant video system at and stored in an encrypted secure server; 50% of cases (*n* = 7 groups of PED and 7 groups of FFCT) will be reviewed after each session using a fidelity checklist. To minimize drift, the PI and project manager will review a random sample of 25% of FFCT cases (*n* = 4 groups) and 25% of PED cases (n = 4 groups) for each therapy team (2 therapists/team) to provide ongoing feedback. The clinical supervisor, PI, and project manager will view the video-recorded sessions and use the fidelity treatment checklists to monitor treatment integrity and dosage. After the PI and project manager review sessions each week, they will share feedback immediately with the clinical supervisor and 3 therapists and at monthly meetings with the intervention team to direct restraining efforts as needed.

#### FFCT/PED adherence assessment and analysis

In years 4 and 5, two external adherence raters will be trained to code adherence on a random sample of 25% of video- recorded cases for the FFCT and PED treatment, even though PI and supervisor ratings will occur throughout the trial. The two adherence training manuals and the rating scales from both treatments will be integrated into one manual and one adherence measure. Two raters who will be blind to the aims of the study will also be trained. Twenty-five percent of cases (4 FFCT groups, 5 sessions/group × 4 groups = 20 sessions and 4 PED groups, 5 sessions/group × 4 groups = 20 sessions) will be randomly selected from each treatment condition. Adherence raters will be 2 undergraduate psychology students recruited from area universities. In order to train the raters (20 h of required training), the integrated manuals and scales will be given to them to read thoroughly. They will also read excerpts from pilot cases that reflect low, medium, and high extensiveness for each of the adherence items. In addition, the raters will review practice videos, also from the pilot. The raters will meet with the team weekly to discuss, as a group, their scores and questions about items, as well as resolve scoring discrepancies. We will derive inter-rater reliability through interclass correlation coefficients (ICC) per Shrout and Fleiss [33] through variance components models, assessing variability between the two raters compared to the total variability in adherence scores. Once the raters have obtained adequate inter-rater reliability with evaluation team and among themselves (ICC > .70) for each item, they will rate actual sessions from the study. Analysis will then focus on whether the therapists delivered the two treatments as intended.

#### Study measures/assessments

This study uses valid and reliable measures, all of which were used in our most recent pilot study with AA families, where there was no evidence or report of respondent burden or fatigue. The assessment schedule appears in Table 1.

**Table 1:** Assessment Schedule

	*Source*	*Wk 0*	*Wk 8*	*Wk 24*	*Wk 52*
*General and Program Descriptive*					
*Demographic Family Survey*	*Parent*	*∙*			
*Consumer Satisfaction*	*Adol/Parent*		*∙*		
*Adolescent Psychological Outcomes*					
*Child Depression Inventory (CDI-2)*	*Adol*	*∙*	*∙*	*∙*	*∙*
*Revised Child Manifest Anxiety Scale (RCMAS-2)*	*Adol*	*∙*	*∙*	*∙*	*∙*
*Parent Psychological Outcomes*	*Parent*	*∙*	*∙*	*∙*	*∙*
*State-Trait Anxiety Inventory (STAI)*	*Parent*	*∙*	*∙*	*∙*	*∙*
*Center Epidemiological Depression Scale (CES-D)*	*Parent*	*∙*	*∙*	*∙*	*∙*
*The Functional Assessment of Cancer Therapy FACT-G (version 4)*	*Parent*	*∙*	*∙*	*∙*	*∙*
*Parent-Adolescent Relational Outcomes*					
*Security Scale*	*Adol*	*∙*	*∙*	*∙*	*∙*
*Short Form Interaction Behavior Questionnaire (IBQ)*	*Adol/Parent*	*∙*	*∙*	*∙*	*∙*
*General Communication*	*Adol/Parent*	*∙*	*∙*	*∙*	*∙*
*Treatment Moderators*					
*Parenting Concerns Questionnaire (PCQ)*	*Parent*	*∙*	*∙*	*∙*	*∙*
*Helpful Group Experiences (HGE)*	*Parent*		*∙*		

##### Adolescent psychological outcomes

ᅟ

#### Depression

The most widely used self-report measure is the *Children’s Depression Inventory (CDI-2)*, which was adapted from the Beck Depression Inventory (BDI) and is reliable and valid for children 7 to 18 years of age [34]. The CDI-2 includes 28 items that assess behaviors associated with depression (e.g., sleep disturbance, anhedonia, suicidality, and appetite loss). Items are rated on a 3-point scale. Raw scores are converted to T scores. T-scores of 70 and higher on the total 28-item CDI-2 scale indicate severe symptoms of depression and is an exclusion criterion. For this study, a cutoff score of 70+ or higher on the T-score will be used to screen out AA adolescents because it indicates severe levels of depressive symptoms [35, 36].

#### Anxiety

The *Revised Children’s Manifest Anxiety Scale (RCMAS-2)* includes 49 items, rated as true or not [37]. The *RCMAS-2 yields scores for six scales*: 1) *Inconsistent Response* (INC; 9 pairs) index—new; 2) *Defensiveness* (DEF; 9 pairs)—renamed; was Lie. 4 Anxiety scales: 3) *Total Anxiety* (TOT; 40 items); 4) *Physiological Anxiety* (PHY; 12 items); 5) *Worry* (WOR; 16 items)—renamed—was Worry/oversensitivity; and 6) *Social Anxiety* (SOC; 12 items)—renamed—was Social Concerns/Concentration. Extensive reliability (α = .85 and adequate internal consistency), validity (e.g., correlation of .85 with State-Trait Anxiety Inventory [STAI] for Children and significant correlation with teacher observations), and normative data have been documented. A cutoff score of 71 (T-score) or higher will be used to screen out AA adolescents with severe anxiety [37].

##### Adult psychological outcomes

ᅟ

#### Anxiety

The *State-Trait Anxiety Inventory (STAI)* will be used to measure anxiety [38, 39]. It includes 40 items (4-point Likert scale), approximately half of which are worded positively (e.g., “I feel regretful” about anxiety that is present) and the other half, negatively (e.g., “I feel pleasant” about anxiety that is absent). The STAI is a reliable and valid measure of state and trait anxiety [38].

#### Depression

The *Center for Epidemiological Studies Depression Scale (CES-D)* is a widely used, 20-item self-report scale that measures the current level of depressive symptoms in the general population, with an emphasis on depressed mood during the past week [40]. Scores range from 0 to 60, with higher scores indicating more symptoms of depression. CES-D scores of 16 to 26 are considered indicative of mild depression, and scores of 27 or more indicate major depression among medical patients.

#### Quality of life

The *Functional Assessment of Cancer Therapy (FACT-G, version 4)* is a brief self-report measure of general cancer quality of life for patients with any tumor type receiving cancer treatment [41, 42] The 27 items assess physical well-being, social/family well-being, emotional well-being, functional well-being, and additional concerns during the past 7 days. Items are rated on a 5-point Likert scale (0=“Not at all” to 4 = “Very much”). The FACT-G demonstrates ease of administration, brevity, reliability, validity, and sensitivity to change [42].

##### Parent-adolescent relational outcomes (mediators)

ᅟ

#### Attachment

The *Security Scale* is a 15-item measure that assesses children’s perceptions of attachment during middle childhood and adolescence [43]. This scale provides a continuous measure of security, evaluating a child’s belief in the responsiveness and availability of the attachment figure, the child’s use of the attachment figure as a safe haven, and the child’s report of open communication with the attachment figure. The Security Scale presents children with descriptions of two types of children and asks which type of child they are most like. Items are scored from 1 to 4, with greater attachment security represented by a higher score. Scores on the Security Scale have adequate internal consistency and evidence of validity based on security scores correlated with self-esteem, peer acceptance, behavioral conduct, physical appearance, and scholastic competence [44].

#### Parent-child relationship

The *Interaction Behavior Questionnaire (IBQ)* is a valid and reliable measure that evaluates agreement and conflict in parent-child dyads [45]. The original IBQ was adapted into a short form consisting of 20 true/false items assessing communication-conflict behavior. Higher scores indicate a more positive relationship.

#### General communication

In this research, 10 questions will be asked to measure general communication between a parent and his/her adolescent child [46]. Each question is scored on a 4-point Likert scale ranging from 1 (strongly disagree) to 4 (strongly agree). It is a valid and reliable measure; higher scores indicate better communication.

#### Potential treatment moderators

At baseline, parents will provide information about their adolescent child’s age and gender. They will also be asked to report household income, education, and family size, age, gender, marital status, cancer diagnosis and stage, time since diagnosis, treatment modalities and frequencies, and mental health diagnoses and treatment.

#### Parental stress

The 15-item (Likert scale) *Parenting Concerns Questionnaire (PCQ)* has good internal consistency (α = .83) and is a valid measure of parenting distress among cancer patients [47]. There are 3 subscales and a total scale, with higher scores indicating more parental stress: (1) practical impact of illness on child; (2) emotional impact of illness on child; (3) concerns about co-parent. PCQ scores are significantly associated (*P* < .01) with standardized measures of psychosocial distress (Distress Thermometer, Hospital and Anxiety Depression Scale, and FACT-G) in the expected directions.

#### Perceived level of group support

The *Helpful Group Experiences (HGE)* is a 17-item adapted valid and reliable measure that assesses the helpfulness of experiences in a support group [48, 49]. The HGE includes 5, four point-subscales, including altruism (e.g., “I offered empathy or compassion”), cognitive growth, and informational (e.g., gained access to important information) support (e.g., got support and encouragement). The HGE will be used only at post-intervention (week 8) to assess parents’ perceptions of group experiences. The 5 subscales have acceptable internal consistency ranging from α = .71–.93 [49].

### Satisfaction with intervention

#### Consumer satisfaction

Parent and Adolescent Consumer Satisfaction includes several Likert and open-ended questions to assess satisfaction with an intervention program, skills taught, teaching methods, and group facilitators [50]. This tool will be used only at post-intervention (week 8) and has been used successfully in parenting intervention research and in two previous pilot studies [8, 27, 28].

### Analysis plan

#### Primary aim 1

Compare the efficacy of FFCT to PED in reducing child depressive symptoms (CDI) in AA adolescents at post-treatment using an intention-to-treat (ITT) analysis. ANCOVA is the most powerful test of the primary study hypothesis and will be estimated by an equation such as:

$$ CDI{(Post)}_i={\beta}_0+{\beta}_1\times CDI{(Pre)}_i+{\beta}_2\times FFC{T}_i+{\sum}_{\mathrm{j}=1}^{\mathrm{J}}{\beta}_j Co{v}_{ji}+{\epsilon}_i $$

Evidence in favor of the study hypothesis will be indicated by a significant negative coefficient for β_2_. In addition to the main effects shown here, we will evaluate the assumption of parallel slopes by a baseline×FFCT interaction. We will evaluate sensitivity of study conclusions to missing data using a range of suitable ITT methods (e.g., last observation carried forward, direct maximum likelihood, multiple imputations). Methods for non-ignorable nonresponse (pattern mixture models) will also be evaluated as indicated. Significant treatment differences at posttest will be followed by subsample analyses to determine individual characteristics associated with differences in treatment effects (e.g., heterogeneity). Standard errors will adjust for clustering within site and family via the cluster-adjusted sandwich estimator.

#### Secondary aim 2

Compare the efficacy of FFCT to PED in reducing parental stress (PCQ) in AA parents at post-treatment using ITT analysis. We will evaluate the secondary aim for posttest differences in parental stress (PCQ) in a fashion parallel to that used for the Aim 1, using an equation such as:

$$ PCQ{(Post)}_i={\beta}_0+{\beta}_1\times PCQ{(Pre)}_i+{\beta}_2\times FFC{T}_i+{\sum}_{\mathrm{j}=1}^{\mathrm{J}}{\beta}_j Co{v}_{ji}+{\epsilon}_i $$

Evidence in favor of the study hypothesis will be indicated by a significant negative coefficient for β_2_.

#### Exploratory aim 3a

Determine trajectories of child depressive symptoms (CDI), anxiety (RCMAS), and parental stress (PCQ) from baseline to 12-month follow-up. Aim 3a will initially be evaluated using random coefficients models using equations such as:

$$ CD{I}_{ij k}=\left({\beta}_0+{\beta}_{0i}+{\beta}_{0k}\right)+\left({\beta}_1+{\beta}_{1i}+{\beta}_{1k}\right)\times Tim{e}_{ij}+{\sum}_{\mathrm{j}=1}^{\mathrm{J}}{\beta}_j Co{v}_{ji}+{\epsilon}_i $$

where coefficients without subscripts represent fixed effects, coefficients indexed by i represent individual-level random effects, subscripts indexed by j represent time effects, and subscripts indexed by k represent treatment group differences. Evidence in favor of our hypothesis would be indicated by a significant negative coefficient for β_1k_ for CDI, RCMAS, and PCQ.

#### Exploratory aim 3b

Determine whether perceived group support, adolescent gender and age, parent’s marital and socioeconomic status, and parent’s cancer staging modify the effects of treatment on child depression and anxiety. We will extend the models estimated above to evaluate key theoretically derived interaction terms. Predicted values will be plotted and, where appropriate, effect modifiers will be dichotomized to capture the region of effect modification. We will estimate standard errors and confidence intervals using bootstrap replications when indicated. Prior research with predominantly white samples suggests the intervention will reduce child depression and anxiety and parental stress to a greater extent for parents perceiving more group support, parents who are single and of low socioeconomic status, adolescent girls, older adolescents, and parents with more severe stages of solid tumor cancer.

#### Exploratory aim 3c

Determine whether pre-post changes in parent-adolescent attachment and communication mediate the association between treatment and adolescent depressive symptoms (CDI) and anxiety (RCMAS) at 6- and 12-month follow-ups. Consistent with current recommended practice, potential mediators will be estimated using structural equation modeling with standard errors estimated using a large (e.g., 5000) number of replicate bootstrap samples. Variance will be partitioned into direct, indirect, and total effects for interpretation. Evidence for mediation is indicated by significant indirect effects. In addition to significance testing, we will evaluate these exploratory aims from an accuracy in parameter estimation (AIPE) framework to bracket estimated effect sizes using half-widths.

### Statistical methods

All digital and hard copies of data will be kept at University of Delaware in a locked lab and stored on a secure server. Within this room are encrypted computers and lockable file cabinets for the PI to store his electronic and hard copies of any data. All information collected will be coded with a study identification number and stored in a locked file cabinet in a locked office, and only staff associated with the study will have access to it. A list of patient names corresponding to study identification numbers will be kept in a password protected file on a secure server in accordance with the standards set forth by the American Psychological Association and the American Psychiatric Association. These records are protected by the confidentiality system of University of Delaware and will not be disclosed except as required by law.

All correspondence that will be mailed to participants will be enclosed in a sealed envelope and will not include identifying information in the return address on the envelope. A full copy of the data set will be shared with only research staff housed at the University of Delaware. All other data sets will be completely de-identified. Electronic files will be encrypted and identified by study specific identifiers (e.g., study ID #s) only. Video recorded therapy sessions will be transferred by the following mechanisms: encrypted flash drive or encrypted portable hard drive; CD ROM; Secure FTP. Any flash drives or other removable media uses will be encrypted. Prior to sending, the dataset will be coded and saved in a password protected files stored on a password protected. Storage of all data at will follow the same confidentiality procedures and precautions taken at University of Delaware.

### Data safety monitoring board/DMC

This randomized controlled trial will be monitored by a Data Safety Monitoring Board/DMC. Given the documented distress of families coping with solid tumor parental cancer, ensuring the highest quality of data gathering and monitoring procedures is essential and enhanced by the monitoring of the study by outside personnel. A detailed Data Safety Monitoring Plan will be submitted to University of Delaware’s Office of Regulatory Affairs, NIH and the Office of Human Subject Protection.

The DSMB will meet twice per year either in-person or via conference call. Board members must be familiar with the protocol and study procedures. The DSMB will review the study protocol and plans for data and safety monitoring (including the reporting of adverse events to the IRBs, and NIH as described below) and consumer confidentiality prior to the start of data collection. After the initiation of the study, the DSMB will be responsible for evaluating the progress of the trial, including periodic assessments of participant recruitment and retention, risks versus benefits to participants, and new developments that are relevant to the participants’ safety or the ethics of the study. In addition, the DSMB will be responsible for providing feedback and suggestions to the PI about these issues, and for recommending whether the study should be continued or terminated.

## Discussion

Oncology support is available for these patients diagnosed with cancer but limited resources for the children of parents with cancer [22]. African Americans parents experience solid tumor cancer at increased rates compared with Whites, putting their school-aged children at higher risk for emotional distress. However, there are few culturally adapted treatments to assist African American adolescents cope with parental cancer. Historically, African Americans with parental cancer tend to take their stress and concerns to spiritual community members, relying on their faith in God [15]. We have developed a culturally adapted family intervention to facilitate family resilience [8, 27]. This RCT will be the first study to implement this culturally adapted family intervention with African American parents with a solid tumor cancer diagnosis parenting school-aged adolescents. We aim to improve parent-child communication by facilitating opportunities to have candid conversations about the cancer diagnosis, treatment and discuss emotional reactions during the intervention group in a supportive environment. This RCT addresses several treatment barriers (e.g., transportation, child care) to aid in recruitment and retention of these families [15].

## Abbreviations

AA: African American
ACS: American Cancer Society
AIPE: Accuracy in Parameter Estimation
ANCOVA: Analysis of Covariance
BDI: Beck Depression Inventory
CCHS: Christiana Care Health Systems
CDI: Children’s Depression Inventory
FACT-G: Functional Assessment of Cancer Therapy
FFCT: Families Fighting Cancer Together
HGE: Helpful Group Experiences
HIPPA: The Health Insurance Portability and Accountability Act of 1996
ITT: Intention To Treat
PCQ: Psychological Capital Questionnaire
PED: Psychoeducation
PI: Principal Investigator
RCMAS: Revised Children’s Manifest Anxiety Scale
RCT: Randomized Control Trial
SAS: Statistical Analysis System
SPSS: Statistical Package for the Social Sciences
STAI: State-Trait Anxiety Inventory
